# Characterization of the complete mitochondrial genome of the green alga *Graesiella emersonii* GEGS21 (Chlorophyta)

**DOI:** 10.1080/23802359.2025.2595395

**Published:** 2025-12-16

**Authors:** Nam Seon Kang, Chang Rak Jo, Myung-Hwa Shin, Kichul Cho, Biet Thanh Tran, Jung Soo Heo, Keun-Yong Kim, Hyung June Kim

**Affiliations:** ^a^ National Marine Biodiversity Institute of Korea, Seocheon, Republic of Korea; ^b^ Bioinformatics Team, AquaGenTech Co., Ltd, Busan, Republic of Korea

**Keywords:** Mitogenome, phylogeny, microalgae, chlorophyta, *Graesiella emersonii*

## Abstract

*Graesiella emersonii* is a green microalga, belonging to the phylum Chlorophyta, with ecological and industrial significance. However, its phylogenetic position and mitogenome structure remain largely unexplored. We analyzed the complete mitogenome of *G. emersonii* GEGS21. This genome was 35,893 bp in length and contained 43 genes that comprised 13 protein-coding, six ribosomal RNA, and 24 transfer RNA genes. Phylogenetic analysis based on six protein-coding genes placed *G. emersonii* GEGS21 in a strongly supported clade with *Pectinodesmus pectinatus*, confirming its affiliation within the order Sphaeropleales. This study will provide valuable insights into the mitogenome architecture of *G. emersonii*, thereby contributing to further elucidation of green algal phylogeny and organelle genomic evolution.

## 1. Introduction

The genus *Graesiella*, belonging to the phylum Chlorophyta, was first established by Kalina and Puncochárová (1987) with *Graesiella vacuolata* as the type species. Later, *G. vacuolata* was treated as a taxonomic synonym of *Graesiella emersonii* (Shihira & Krauss) Nozaki et al. 1995 (Nozaki et al. 1995). Thus, *G. emersonii* is currently recognized as the only accepted species within the genus (Guiry and Guiry 2025). This green alga has often been observed in brackish waters (Robinson 1998; Kang et al. 2022). This species is a promising biological resource, owing to its ability to produce valuable pigments, high-value lipids, and bioactive compounds (Sawant and Mane 2018; Desai et al. 2020; Perdana et al. 2021; Kumar et al. 2022).

Despite its industrial significances, the phylogenetic position and genomic structure of *G. emersonii* remain largely unexplored. Previous studies have primarily focused on physiological traits and phylogenetic analyses based on DNA barcoding markers rather than the genomes of its organelles (Kang et al. 2022). In this study, we report the complete mitochondrial genome (mitogenome) of *G. emersonii* for the first time.

## 2. Materials and methods

### 2.1. Algal culture and genomic DNA extraction

**Figure 1.**
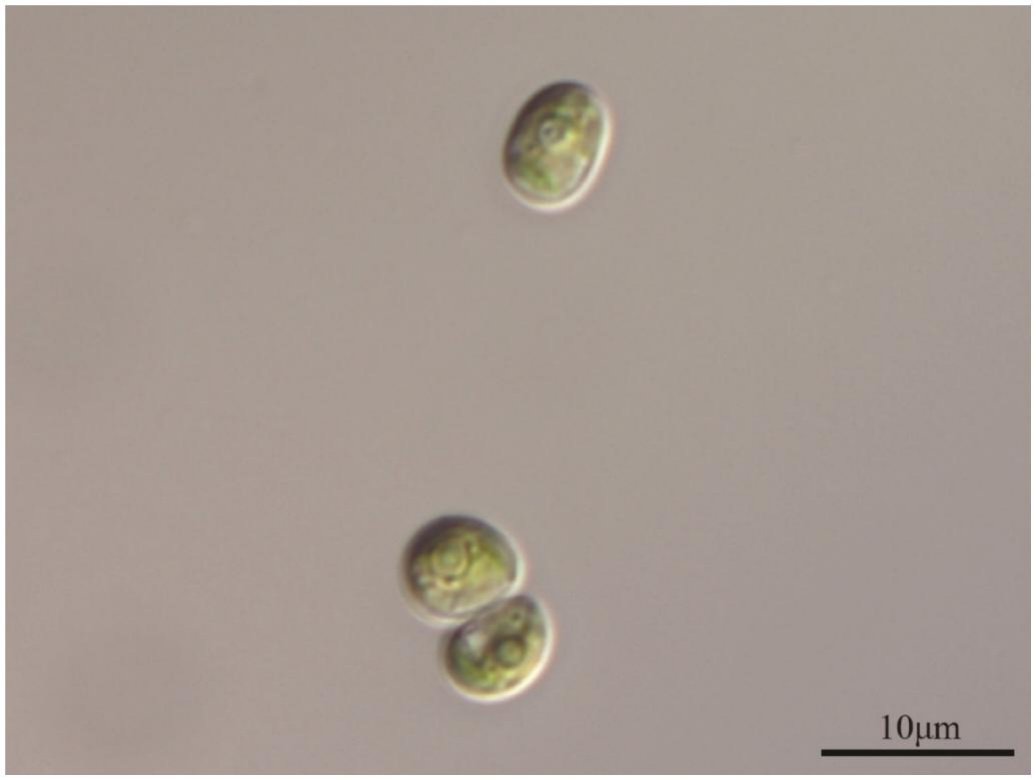
Specimen image of *Graesiella emersonii* GEGS21.

An algal strain, *G. emersonii* GEGS21 was isolated from the Geumgang Estuary, located in Gunsan-si, Jeollabuk-do, Korea (36°08′20.0″N, 126°45′10.8″E) in May 2021. A specimen was deposited at the National Marine Biodiversity Institute of Korea (MABIK; https://www.mabik.re.kr), contact: Dr. Nam Seon Kang (email: kang3610@mabik.re.kr), under the voucher number MABIK LP00000155 (Figure 1).

The strain was identified based on morphological features observed under an Olympus BX53 light microscope (Olympus, Tokyo, Japan), including ellipsoidal cell shape, a single parietal chloroplast with a pyrenoid, and a smooth cell surface, consistent with the description by Nozaki et al. (1995). In addition, a phylogenetic tree based on 18S + ITS rDNA sequences (Supplementary Figure S1) confirmed that GEGS21 clustered with authentic strains of *G. emersonii*.

Total genomic DNA was extracted from 100 mL of its algal culture after centrifugation at 3,000×*g* using the APBIO APrep™ Plant DNA Kit (APBIO Co., Namyangju-si, South Korea). Its quality was assessed using a NanoDrop One Microvolume UV-Vis Spectrophotometer (Thermo Fisher Scientific Inc., Wilmington, DE, USA) and Quant-iT™ PicoGreen™ dsDNA Assay Kit (Thermo Fisher Scientific Inc.).

### 2.2. Sequencing and data processing

A library for next-generation sequencing (NGS) analysis was prepared using the xGen™ DNA Library Preparation Kit (Integrated DNA Technologies, Skokie, IL, USA) for paired-end sequencing (2 × 150 bp) on the NovaSeq™ 6000 Sequencing Systems (Illumina, San Diego, CA, USA). Raw sequencing reads produced by the NGS analysis were processed using Cutadapt v3.11 (Martin 2011). Adapter sequences were trimmed, followed by filtering of low-quality reads (*Q* < 20) and removal of reads shorter than 50 bp. Cleaned reads were *de novo* assembled using the CLC Genomics Workbench (CLC bio, Aarhus, Denmark) with similarity and length fractions of 0.8 and 0.5, respectively.

### 2.3. Mitogenome analysis and annotation

The assembled contigs were subjected to a BLASTN search (E-value = 0.001) implemented in the CLC Genomics Workbench against the NCBI nucleotide database (National Center for Biotechnology Information 1988). Contigs with hits to mitochondrial genes were extracted and extended using cleaned reads in Geneious v2021.1.1 (Biomatters Inc., Auckland, New Zealand). Genes were annotated using BLASTN, BLASTX, and Chloe v0.1.0 in the GeSeq webserver (Tillich et al. 2017). The draft mitogenome was used as a reference to map the cleaned reads and read depth coverage was calculated in Geneious v2021.1.1 based on the number of reads mapped per nucleotide position; the mean coverage across the assembly was obtained from the mapping output. A mitogenome map was generated using OGDRAW v1.3.11 (Greiner et al. 2019). The annotated sequence of its complete mitogenome was deposited in GenBank under accession number PV433165.

### 2.4. Phylogenetic tree construction

To construct the phylogenetic tree of *G. emersonii* GEGS21, ten mitogenome sequences from the order Sphaeropleales and two outgroup species from the order Chlamydomonadales—*Haematococcus lacustris* (NC_044670) and *Chlamydomonas reinhardtii* (NC_001638) belonging to the class Chlorophyceae—were obtained from GenBank database (Table 1). Sequences were aligned using ClustalW in BioEdit v7.2.5 (Hall 1999) with default parameters. A concatenated alignment of six protein-coding genes (*co1*, *cyb*, *nd1*, *nd2*, *nd4*, and *nd6*) was used for phylogenetic analysis. The best-fit model, GTR+G + I, was selected *via* JModelTest v.2 (Darriba et al. 2012). Maximum likelihood (ML) analysis was performed using RAxML v.7.0.4 (Stamatakis 2006; Stamatakis et al. 2008) with 1,000 bootstrap replicates. Bayesian inference (BI) was conducted in MrBayes v.3.1.2 (Ronquist et al. 2012) with 1,000,000 generations, discarding the first 25% as burn-in once the average standard deviation of split frequencies fell below 0.01. The resulting tree was visualized using TreeViewX v.0.5.0 (Page RDM 2002).

**Table 1.** List of accession number and publication used in the phylogenetic tree.

Order	Species	GenBnank acc. no.	Reference
Sphaeropleales	*Ankistrodesmus falcatus*	NC_051461	Cobos et al. 2021
	*Bracteacoccus aerius*	NC_024755	Fučíková et al. 2014
	*Bracteacoccus minor*	NC_024756	Fučíková et al. 2014
	*Chlorotetraedron incus*	NC_024757	Fučíková et al. 2014
	*Chromochloris zofingiensis*	NC_024758	Fučíková et al. 2014
	*Gormaniella terricola*	NC_066824	Robison et al. 2022
	*Graesiella emersonii*	PV433165	This study
	*Jenufa perforata*	NC_046779	Turmel et al. 2020
	*Mychonastes homosphaera*	NC_024760	Fučíková et al. 2014
	*Ourococcus multisporus*	NC_024762	Fučíková et al. 2014
	*Pectinodesmus pectinatus*	NC_036659	Zhao et al. 2022
Chlamydomonadales (outgroup)	*Chlamydomonas reinhardtii*	NC_001638	Vahrenholz et al. 1993
	*Haematococcus lacustris*	NC_044670	Zhang et al. 2019

## 3. Results

**Figure 2.**
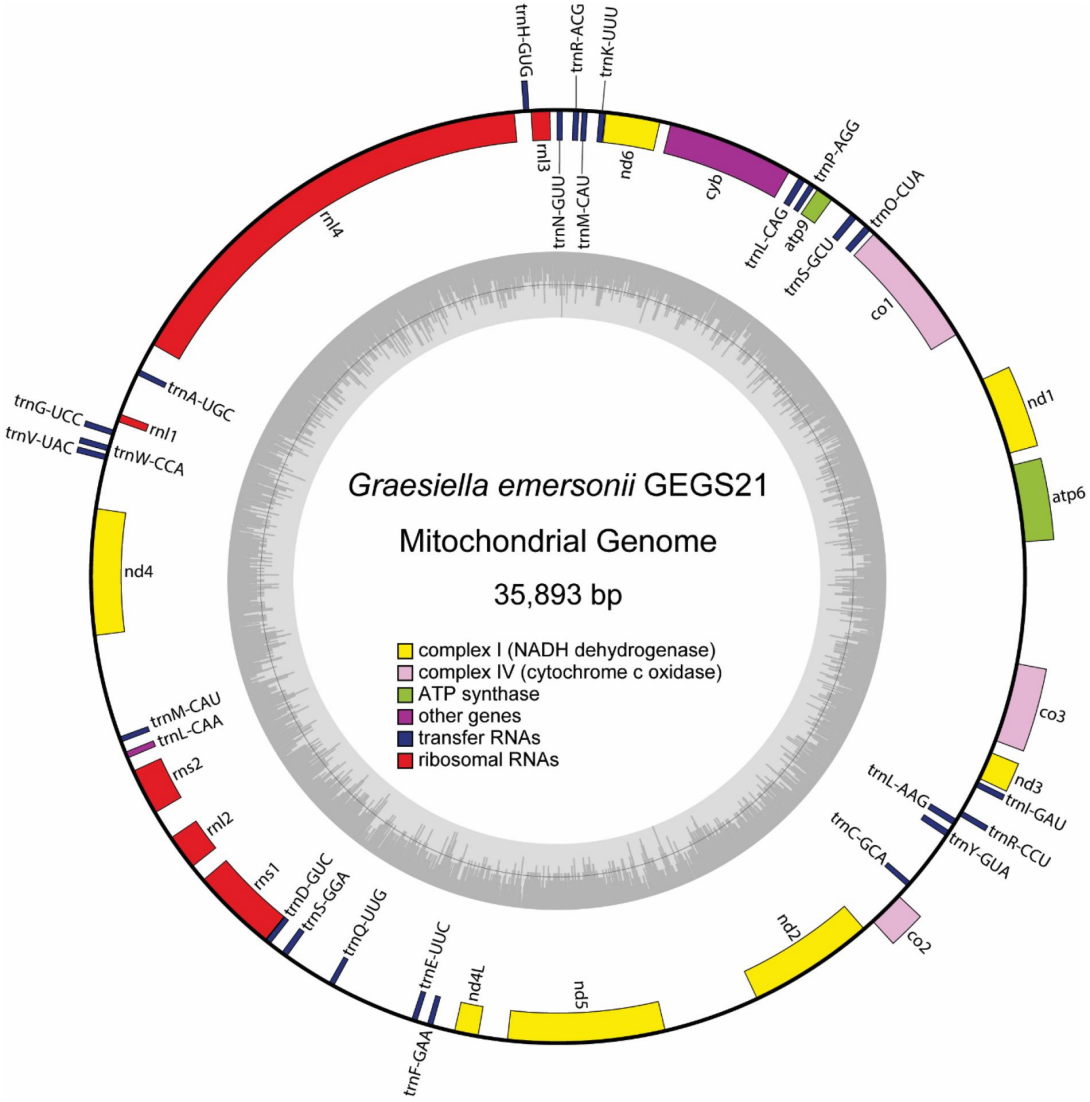
The circular mitogenome map of *Graesiella emersonii* GEGS21 (GenBank accession number PV433165). Genes encoded on the reverse and forward strands are illustrated inside the circle and outside the circle, respectively.

The NGS analysis of *G. emersonii* GEGS21 yielded 11,300,000,000 bp from 37,389,275 reads. Its genomic composition showed a nearly balanced nucleotide distribution, with 50.7% GC content and 49.3% AT content. The sequencing quality was notably high, with 97.1% of bases scoring ≥ Q20 (1 in 100 probability of an incorrect base call) and 93.7% scoring ≥ Q30 (1 in 1000 probability of an incorrect base call). The complete *G. emersonii* mitogenome was characterized as a circular, double-stranded DNA molecule comprising 35,893 bp (Figure 2). It contained a total of 43 genes composed of 13 protein-coding genes (PCGs), and six ribosomal RNA (rRNA) and 24 transfer RNA (tRNA) genes. The clean raw sequencing data mapped to this mitogenome, achieving 100% coverage with a minimum depth of 7,329×, an average depth of 13,100×, and a maximum depth of 19,164× (Supplementary Figure S2).

The PCGs included key components of the oxidative phosphorylation pathway: *atp6*, *atp9*, *co1–3*, *cyb*, and *nd1–6*. All PCGs used ATG as the start codon, whereas the stop codons varied, with TCA being predominant (used in ten genes) and TGA occurring in *atp6* and *nd6*. The rRNA genes comprised four and two copies of the 23S and 16S rRNA genes, respectively, primarily located on the reverse strand. The 24 tRNA genes encoded all 20 standard amino acids, with *trnL*, *trnM*, and *trnS* appearing twice. The *cyb* gene is interrupted by a 379 bp intron (Supplementary Figure S3**).**

**Figure 3.**
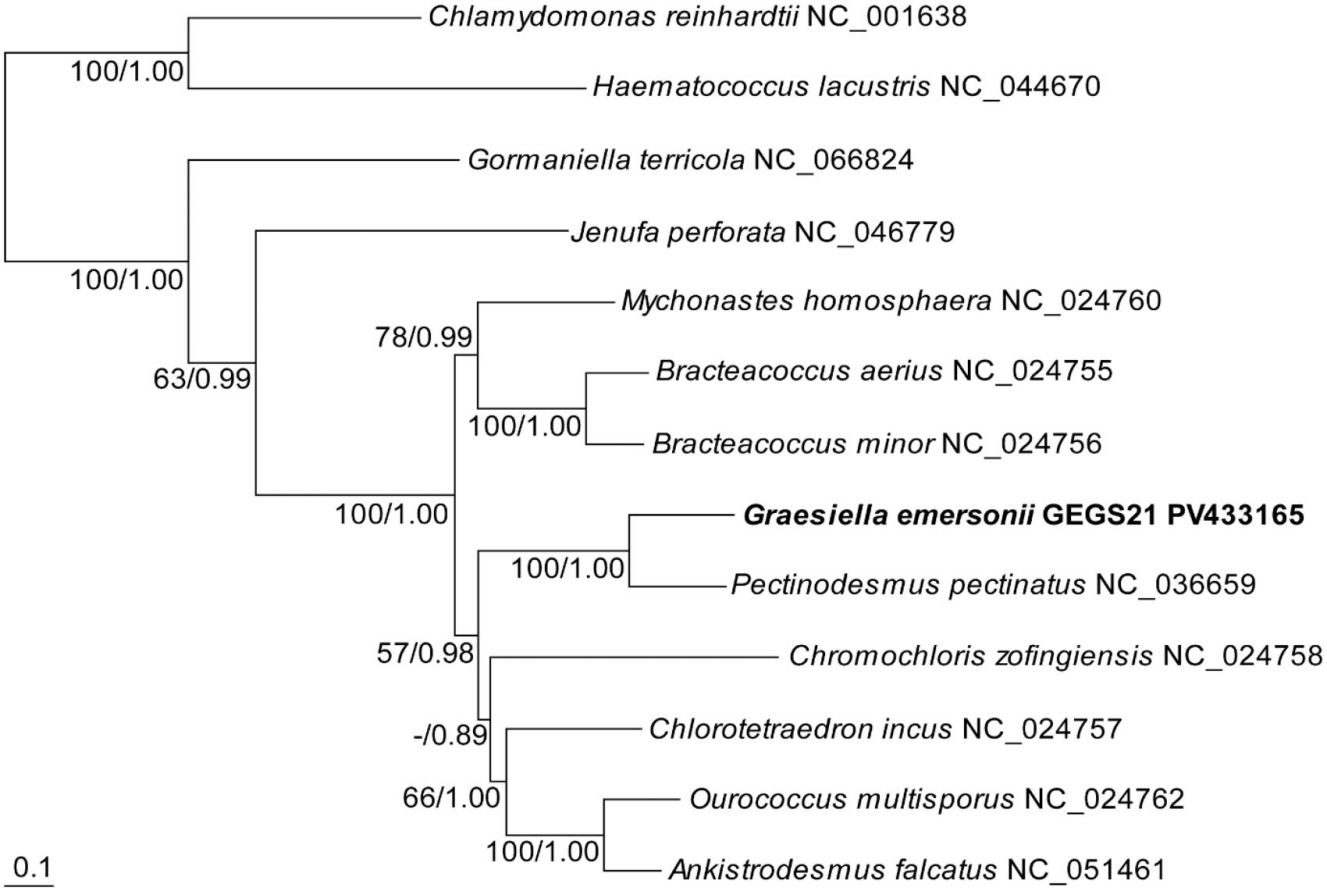
Maximum likelihood (ML) phylogenetic tree inferred from concatenated nucleotide sequences of six mitochondrial protein-coding genes (*co1*, *cyb*, *nd1*, *nd2*, *nd4*, and *nd6*) obtained from complete mitogenomes of species within the order sphaeropleales. Support values at each node indicate ML bootstrap percentages (above 50%) followed by bayesian posterior probabilities.

The phylogenetic analysis based on six mitochondrial PCGs placed *G. emersonii* GEGS21 in a strongly supported clade with *P. pectinatus* (ML bootstrap 100%; BI posterior probability 1.00), confirming its affiliation within the Sphaeropleales (Figure 3, Table 1).

## 4. Discussion and conclusion

This study presents the first complete mitogenome of *G. emersonii* GEGS21. The mitogenome is a circular DNA molecule of 35,893 bp, comprising 43 genes—13 PCSs, six rRNA, and 24 tRNA genes—with a balanced GC content of 50.7%. Its gene content and arrangement are consistent with other members of the order Sphaeropleales belonging to the class Chlorophyceae, particularly in components of the oxidative phosphorylation pathway. The mitogenome structure aligns with those of related green algal taxa, supporting a conserved genomic architecture within the Sphaeropleales belonging to the Chlorophyceae (Fučíková et al. 2014; Zhao et al. 2022). In *G. emersonii*, all PCGs initiated with the canonical start codon ATG, and most terminated with TCA. This pattern is consistent with *P. pectinatus* (Zhao et al. 2022), *Tetradesmus obliquus* (Nedelcu et al. 2000), and other species of the Sphaeropleales (Fučíková et al. 2014). However, TGA is also used as a stop codon in *atp6* and *nd6* in the *G. emersonii* mitogenome. Such mixed stop codon usage is not unusual within the Chlorophyceae, where even closely related taxa can differ markedly (Fučíková et al. 2014). These findings highlight the flexibility of mitochondrial translation termination in green algae and the evolutionary plasticity of the genetic code within the Sphaeropleales.

Phylogenetic analysis based on six mitochondrial PCGs places *G. emersonii* firmly within the Sphaeropleales. While GenBank currently classifies this species under Trebouxiophyceae, both our results and the taxonomic listing in AlgaeBase (Guiry and Guiry 2025) support its placement in the Chlorophyceae. The relationships among the Sphaeropleales taxa in our phylogenetic tree were consistent with previous reports (Fučíková et al. 2014; Zhao et al. 2022), with *G. emersonii* clustering as the closest relative of *P. pectinatus* and other lineages forming the expected clades. Based on this molecular evidence, the GenBank taxonomy for *G. emersonii* should be revised to reflect its true phylogenetic position within the Chlorophyceae. This finding contributes to a more accurate understanding of green algal mitogenome evolution and taxonomy.

## Supplementary Material

Supplementary_Figures_S1–S3_GEGS21.docx

## Data Availability

The mitogenome sequence data that support the findings of this study are openly available in the GenBank database of NCBI under the accession number, PV433165 (https://www.ncbi.nlm.nih.gov/nuccore/PV433165). The associated ‘BioProject,’ SRA, and ‘BioSample’ numbers are PRJNA1240872, SRR32829925, and SAMN47542384, respectively.
