# Supplementary material for: Characterization of the complete mitochondrial genome of the green alga Graesiella emersonii GEGS21 (Chlorophyta)
Source: Mitochondrial DNA B Resour. 2025 Dec 16;11(1):90–4. doi: 10.1080/23802359.2025.2595395 (PMC12710258; doi:10.1080/23802359.2025.2595395)
Supplement: Supplementary_Figures_S1–S3_GEGS21.docx [file TMDN_A_2595395_SM0509.docx]

Supplementary materials

**Characterization of the complete mitochondrial genome of a green alga, *Graesiella emersonii* GEGS21 (Trebouxiophyceae)**

Nam Seon Kang ^a^, Chang Rak Jo ^a^, Myung-Hwa Shin ^a^, Kichul Cho ^a^, Biet Thanh Tran ^b^, Jung Soo Heo ^b^, Keun-Yong Kim ^b^, Hyung June Kim ^a, *^

^a^ National Marine Biodiversity Institute of Korea, Seocheon 33662, Republic of Korea;

^b^ Department of Genetic Analysis, AquaGenTech Co., Ltd, Busan 48228, Republic of Korea

CONTACT Hyung June Kim, E-mail kimhj95@mabik.re.kr National Marine Biodiversity Institute of Korea, Seocheon 33662, Republic of Korea

**
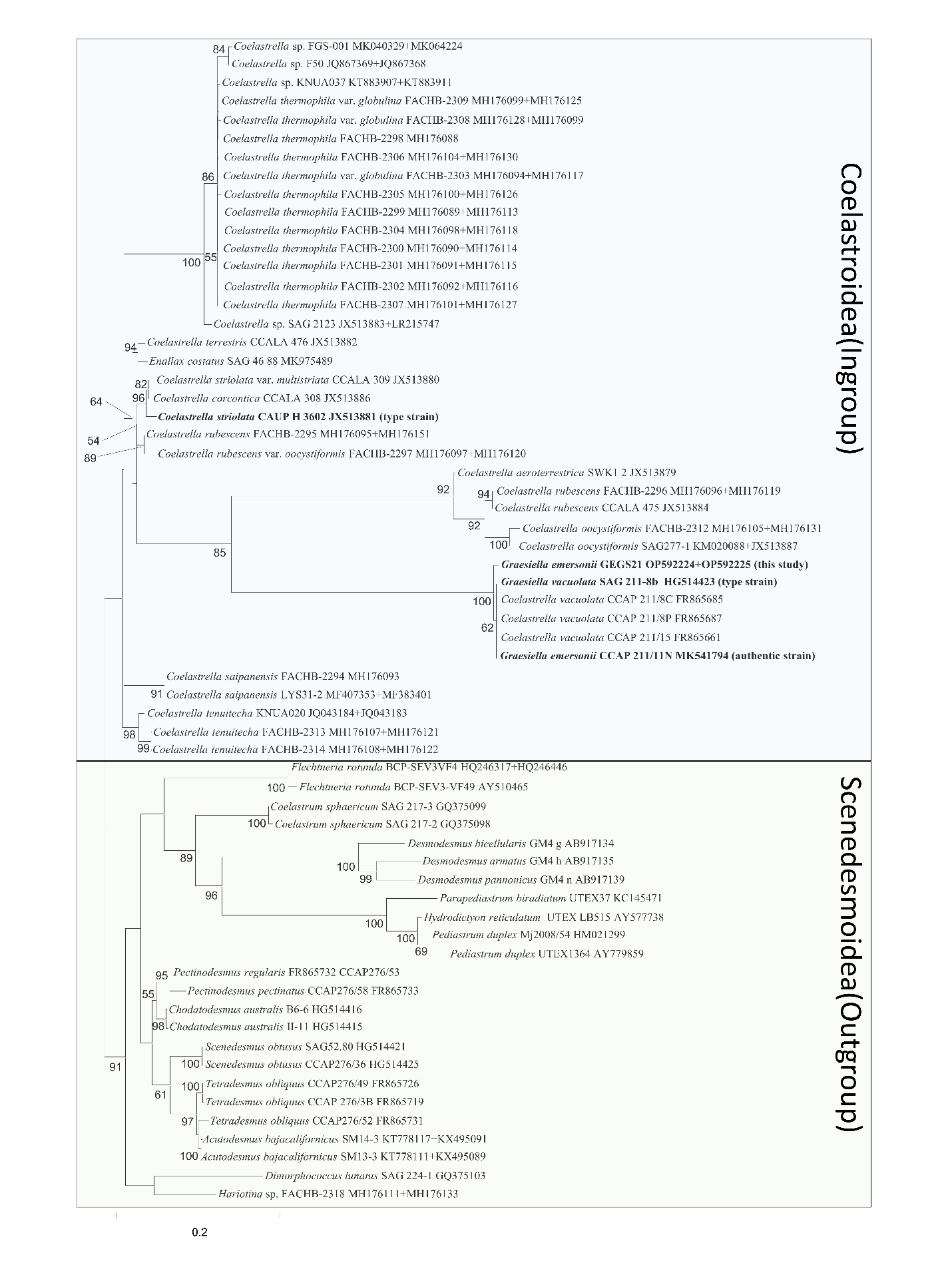
 Figure S1.** Maximum likelihood (ML) phylogenetic tree based on 18S + ITS rDNA sequences reconstructed using raxmlGUI 2.0 under the GTR+G+I substitution model. Bootstrap support values (≥50%) based on 1,000 replicates are shown at the nodes. Taxa belonging to Scenedesmoidea were used as the outgroup. The ingroup (Coelastroidea: Coelastrella + Graesiella) forms a well-supported monophyletic clade. The type strain of Graesiella vacuolata (SAG 211-8b) and the authentic strain of G. emersonii (CCAP 211/11N) are indicated, along with the type strain of Coelastrella striolata. The newly sequenced strain, G. emersonii GEGS21, clusters with authentic G. emersonii strains. The scale bar represents the number of nucleotide substitutions per site.


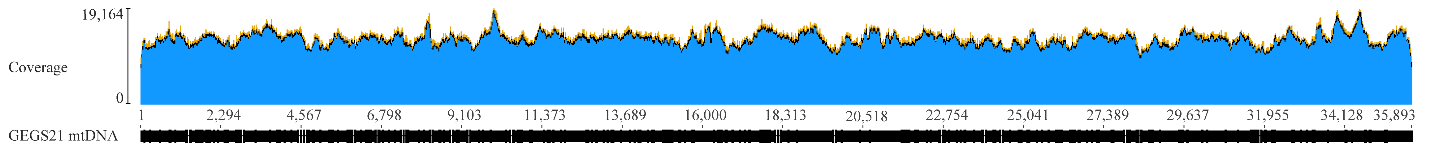
 **Figure S2.** Coverage depth plot of the assembled mitochondrial genome of *Graesiella emersonii* GEGS21 (GenBank accession number PV433165).


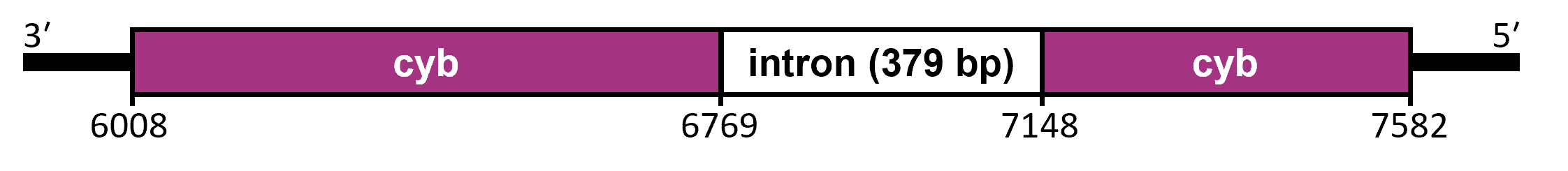
**Figure S3.** Cis-splicing gene map of the cytochrome b (cyb) gene in the mitochondrial genome of Graesiella emersonii GEGS21 (GenBank accession number **PV433165**)*.* The ***cyb*** gene is interrupted by a **379 bp intron** (shown in white), with **two exons** (purple) joined via **cis-splicing**. Nucleotide positions are shown below the diagram.
